# The psychosocial burden of anogenital warts on Syrian patients: study of quality of life

**DOI:** 10.1016/j.heliyon.2022.e09816

**Published:** 2022-06-30

**Authors:** Joud Haddad, Fouz Hasan, Abdel Halim Roumeih, Abdullah Omar

**Affiliations:** a Department of Dermatology, Tishreen University, Latakia, Syria; b Department of Dermatology, Damascus, Syria; c Syrian Private University, Damascus, Syria

**Keywords:** Quality of life, EQ-5D, CECA10, Anogenital warts, HPV

## Abstract

**Purpose:**

The prevalence of anogenital warts caused by HPV has been on the rise in the war-torn country of Syria recently. Although physically mildly symptomatic, this disease has a considerable negative psychological effect on patients. This study showed up to reveal information on the quality of life (QoL) of these patients and its connection to age, gender, disease severity, educational level and marital status.

**Methods:**

The study sample consisted of 57 males and 45 females aged between 18 and 64 years old. The patients were recruited from the main university hospital in Latakia, Syria. The assessment was done using two questionnaires: EQ-5D and CECA10.

**Results:**

The data from the EQ-5D revealed no problems in Mobility, Self-care or Usual Activities. Whereas, our study showed extreme levels of anxiety and depression in roughly 50% of the sample. A noticeable impact was seen in 31% of participants describing moderate pain and discomfort. The CECA10 questionnaire revealed an average of disease-specific QoL of 2.48 which indicates a moderate to severe impact. We noticed a statistically significant relationship between the QoL and the patient's age. The age group of 20–29 has suffered the most. Without statistical significance, the psychological burden was at its worst in single patients with severe disease and a high level of education. Females suffered a slightly worse emotional impact compared to males.

**Conclusion:**

This study proved a negative effect of genital warts on patients mainly young educated individuals, females and severe cases.

## 1 Introduction

An infection transmitted through sexual means and presenting with lesions on private areas is all that it takes to negatively impact the quality of life in people belonging to a conservative society like Syria. Almost 70% of all sexually active individuals will be infected with HPV-caused genital disease at some point of time in their life [1, 2]. Targeting more often young men and women, the Human Papilloma Virus (HPV)-caused anogenital warts have been on the rise in the war-torn country of Syria in the last couple of years. In general, two-thirds of persons who have sexual contact with an infected partner will develop lesions within 1–6 months [3]. Exacerbated by the ongoing conflict, this sexually transmitted infection has been causing a noticeable negative impact on the mental and psychological wellbeing of patients that may range from stress to anxiety up to depression.

Out of 130 HPV types, 40 of them tend to affect the anogenital region [4]. The most common HPV types causing the disease in Europe are types 6 and 11 and are answerable for approximately 90% of the genital wart cases [5, 6] HPV-caused genital warts are one of the most frequently diagnosed STIs in dermatology clinics [7] without clear documentation of the disease incidence and prevalence in Syria. Publications speak of an incidence of over 80000 new cases in 2016 in the UK alone with an estimated peak incidence among 20 to 24-year-old men of 794/100′000 and 767/100′000 in 16–19-year-old women [8]. In the US and Australia, the incidence rate recorded is less than 0.3% [6] and around one million new cases every year in the US [9].

While most infections are asymptomatic, the disease could cause burning and bleeding [9]. The disease course is frequently protracted and recurrent. Relapsing episodes are estimated to occur within 3 months in at least 25% of cases [4, 7, 8].

This study came to tackle the psychological consequences and the direct relationship between getting infected with HPV and the implication on the quality of life. It has been estimated that approximately two-thirds of patients made lifestyle changes regarding sexual relationships after being diagnosed with this disease [3].

The main researcher was in daily contact with anogenital warts patients to provide proper diagnosis and treatment in the vicinity of a large university hospital in the coastal city of Latakia. This city has witnessed minimal physical damage at the beginning of the Syrian armed conflict then has, later on, become the main destination of internal displacement which made it a representative colourful social fabric of all ethnic groups from all around the nation. During this contact with patients, it was clear how this seemingly mild and ‘peaceful’ infection has adversely impacted how patients feel [1, 10, 11].

### 1.1 Objectives

Not performed before in Syria, this study showed up to reveal unprecedented first-hand information on the psychological results of a sexually transmitted infection in both males and females by using 2 standardized questionnaires: EQ-5D and CECA10. In addition, this study compared the relationship between the impact of the disease and the age, gender, disease severity, educational level and marital status.

## 2 Methods

### 2.1 Design

To conduct the cross-sectional study, a random sample of 102 patients (57 males and 45 females, sex ratio 1:1.2) with clinically diagnosed anogenital warts was collected from the Dermatology department of Tishreen University Hospital in Latakia city in Syria. The collection period lasted one year, from November 2016 until October 2017.

### 2.2 Participants

Patients recruited were aged from 18 till 64 years old, diagnosed with anogenital warts, and coming either for a first-time consultation, a follow-up visit, a recurrent episode or a persisting infection more than 3 months. All patients gave verbal consent to voluntarily participate in the study. Patients with concomitant STI, any disease in the genital area or severe systemic or debilitating diseases (paralysis, severe burns, genetic disorders, etc.) were excluded from the sample as these diseases might already affect the quality of life of patients. The study protocol was reviewed and approved by the Head of Dermatology Department and the Faculty of Medicine at Tishreen University.

The demographic information collected was Name, Age, Gender, Educational level and Marital status. The disease severity was one of the essential factors studied and was assessed depending on the number of lesions each patient had. Having 3 lesions or less was classified as Mild while having 4 to 7 lesions was considered a Moderate case compared to Severe cases which indicate patients who have 8 or more visible lesions.

### 2.3 Measures

#### 2.3.1 Quality of life

To assess the impact of anogenital warts on the quality of life of patients, two questionnaires were asked to be filled by each participant. Any questionnaire with un-answered questions was discarded. To increase the study anonymity and to get the participants to feel more comfortable, only the initials were collected in the ‘Name’ field. The majority of patients filled the questionnaires alone after having them explained by the main researcher. Illiterate patients got assistance in reading the questions and filling the answers.

Disease Severity Assessment: To assess the disease severity we counted the number of lesions the patient had at the time of participation in the study.

The objective assessment of the disease severity was clinically done by counting the active lesions. The subjective assessment is - in a way - one of the purposes of the study to figure out eventually how negative the patients perceived it to have a genital infectious disease and to what degree their quality of life was affected.

1. The generic questionnaire: European quality of life index version 5D (EQ-5D): [1, 8].
This generic measure of the quality of life is widely used in economic assessments and it covers 5 dimensions of life (Mobility, Self-care, Usual Activities, Pain/Discomfort and Anxiety/Depression). The participant answers each question by grading the effect on their life as having no, some or extreme problem in that area. In our study, the visual analogue scale (EQ-VAS) part was not used to save patients' time as they had to fill two questionnaires in a social set-up where medical research is not common, especially for such a private disease.
2. Cuestionario Especifico en Condilomas Acuminados (CECA10): [1, 8].
The CECA10 is a disease-specific quality of life assessment, consisting of 10 statements indicating the direct impact of anogenital warts on psychosocial, emotional and sexual wellbeing. The participants estimated the effect of each statement on a 5-grade scale which eventually gives a numeric result and an average. The lower the average, the worse effect is expected on QoL The scaling pattern has been adjusted from a 3 grade-scale in the original questionnaire to a 5-grade scale in our study to give our patients more options to express in detail how they feel in each aspect of life.
Broken down into 3 groups, the first three statements tackled the patient's fear of the disease course and future complications:
  • I am afraid that the lesions won't disappear.
  • I am anxious to know whether I am going to recover from the infection for good.
  • I worry about whether the warts will get worse or whether there will be some complications.
The second group of three statements assessed the level of anxiety and mood alterations:
  • My state of mind is upset (anxiety, depression, sadness, uneasiness…).
  • I feel more insecure.
  • Knowing that I have the illness affects me in my daily life.
And lastly, the 4 statements spoke of the disease effect on the patient's sexual life:
  • My sexual drive has decreased.
  • I feel worried during sexual relations.
  • I avoid sexual relations.
  • My sexual relations have decreased in quality and/or frequency.

#### 2.3.2 Analysis

The statistical analysis used in this study included 2 types of statistics. Firstly, Descriptive statistics where quantitative variables were expressed through the average and the standard deviation (SD) while qualitative variables were converted into percentages. Secondly, Inferential statistics were implemented to study the results and comparisons depending on IBM SPSS.

To measure the internal consistency of our scale, Cronbach's alpha was used to calculate the minimum value needed for the scale to be consistent. This value is accepted when it crosses the threshold of 0.06. For our study, a score of 0.9 was considered sufficient for the scale to be used. The mean time to fill in this questionnaire was 7 min.

Independent T Student and ANOVA analyses were used to compare groups considering a *p-value* of less than 0.05 to be statistically significant. Other statistical methods used were Kruskal – Wallis and Mann Whitney.

## 3 Results

### 3.1 Characteristics of the participants

We can see in Table 1 that the age group of 20–29 years old comprised the majority in the sample with 39.2% while the group of 30–40 years old occupied 30.4% of the sample. In conclusion, the age group of 20–39 years of age was the predominant age frame with around 70% of the study sample. The median age was 31 ± 8.5 years old while the mean was 35 years for males and 28 years for females.

**Table 1:** Demographic profile of respondents.

Age Group (years)	Number of patients	Percentage
Less than 20	2	2%
20–29	40	39.2%
30–39	31	30.4%
40–49	19	18.6%
50–59	7	6.9%
More than 60	3	2.9%
**Educational level**	**Number of patients**	**Percentage**
Illiterate	5	4.9%
Basic school	23	22.5%
High school	35	34.4%
University	39	38.2%
**Marital status**	**Number of patients**	**Percentage**
Single	37	36.2%
Married	63	61.8%
Divorced	2	2%

Looking at the education in Table 1, the group of patients with a university degree represented the most prevalent group of patients with 38% followed by high school degree holders with 34%. It is worth mentioning that by the basic school we meant up to the 9^th^ grade while high school indicated finishing the 12^th^ grade and getting the high school diploma. By university, we meant either university students or degree holders.

The majority of the study sample was married individuals with a percentage of 61.8% followed by single persons with 36.2% as shown in Table 1.

When it comes to disease severity in Table 2, 43% of the sample presented with severe disease of more than 7 lesions, followed by 32% with a moderate disease of 4–7 warts, and 24.5% who had only a mild severity of less than 3 visible papules.

**Table 2:** Distribution of study sample according to disease severity.

Disease severity	Mild	Moderate	Severe	Gender
Number of patients	10	15	32	Males
	15	18	12	Females
Total	25	33	44	
Percentage	24.51%	32.35%	43.13%	

### 3.2 5Q-ED general questionnaire results

After proper collection and analysis, the data from the 5Q-ED revealed no problems reported in Mobility, Self-care or Usual Activities with 86.3%, 87.2% and 89.2% of the sample respectively.

When it comes to pain and discomfort, 20% of the patients reported having an extreme grade of symptoms, 31.4% showing moderate symptoms while 48% reported no pain or discomfort at all.

As for more psychological disturbances of anxiety and depression, 56% of the sample described the extreme degree of these symptoms and 25% described moderate degree while 18.6% stated having no anxiety or depression at all (see Table 3).

**Table 3:** The results of the 5Q-ED questionnaire.

Dimension	Grade of problem	Number	Percentage
Mobility	I have no problems in walking about	88	86.30%
	I have some problems in walking about	14	13.70%
	I am confined to bed	0	0%
Self-care	I have no problems with self-care	89	87.20%
	I have some problems washing or dressing myself	12	11.80%
	I am unable to wash or dress myself	1	01.00%
Usual activities	I have no problems with performing my usual activities	91	89.20%
	I have some problems with performing my usual activities	11	10.80%
	I am unable to perform my usual activities	0	0%
Pain/discomfort	I have no pain or discomfort	49	48.00%
	I have moderate pain or discomfort	32	31.40%
	I have extreme pain or discomfort	21	20.60%
Anxiety/depression	I am not anxious or depressed	19	18.60%
	I am moderately anxious or depressed	26	25.40%
	I am extremely anxious or depressed	57	56.00%

### 3.3 CECA10 specific questionnaire results

To evaluate the overall disease-specific quality of life of patients, we calculated the average score of all answers for all patients. Accordingly, and for our sample, we got the figure of 2.48 which indicates a moderate to severe adverse effect (range from the worst 1 to the best 5).

To estimate the impact of the disease on each aspect of the psychological and sexual wellbeing of patients, we calculated the average points earned for each question. Ranging from 1 to 5, the lower the average, the worse the effect on QoL Based on this, we found out that the worst effect felt by patients with an average of 2.16 was on the state of mind, for instance, the feeling of anxiety, depression and sadness. The second worst effect was seen on the fear of the disease evolution and complications with an average of 2.21. In the third place came the negative effect on the daily life of patients with an average of 2.25. as shown in Table 4.

**Table 4:** The average of each statement of the CECA10 questionnaire.

Statement	Total score	Average
I am afraid that the lesions will not disappear.	272	2.66
I am anxious to know whether I am going to recover from the infection for good.	233	2.28
I worry about whether the warts will get worse or whether there will be some complications.	226	2.21
My state of mind is upset (anxiety, depression, sadness, uneasiness…).	221	2.16
I feel more insecure.	233	2.28
Knowing that I have the illness affects me in my daily life.	230	2.25
My sexual drive has decreased.	327	3.20
I feel worried during sexual relations.	269	2.63
I avoid sexual relations.	266	2.60
My sexual relations have decreased in quality and/or frequency.	260	2.54

We then moved to study the relationship between our different variables and the disease-specific QoL Studying the results using the ANOVA and the Kruskal – Wallis methods revealed no statistically significant relationship between the disease severity, educational level or the marital status and the quality of life with a P-value of 0.1, 0.4 and 0.6 respectively as seen in Tables 5,6, and 7.

**Table 5:** The average quality of life according to disease severity.

Disease severity	Number	Average	SD	P-value
Mild	25	2.9	1.2	0.1
Moderate	33	2.3	1.09	
Severe	44	2.1	1.04

**Table 6:** The average quality of life according to educational level.

Educational level	Number	Average	SD	P-value
Illiterate	5	2.86	1.30	0.4
Basic school	23	2.76	1.26	
High school	35	2.35	1.02	
University	39	2.39	1.06

**Table 7:** The average quality of life according to marital status.

Marital status	Number	Average	SD	P-value
Single	37	2.35	0.84	0.6
Married	63	2.56	1.24	
Divorced	2	2.4	0.98	

However, and with a reasonable clinical significance, we can see that patients with a severe disease scored the least in terms of quality of life effect with an average of 2.1 (SD 1.04) followed by moderate stage with an average of 2.3 (SD 1.09) while the mild disease patients enjoyed the best quality of life with their average of 2.9 (SD 1.2).

Similarly, we noticed that the higher the level of education, the worse quality of life was registered. University graduates and high school diplomas holders had worse quality of life with an average of 2.35 and 2.39 (SD 1.02 and SD 1.06) respectively than illiterate individuals and basic school graduates.

Without a statistical significance, single patients had the worst quality of life with an average of 2.3 (SD 0.84) followed by divorced then married individuals with averages of 2.4 and 2.5 (SD 0.98 and SD 1.24) respectively.

Equally as seen in Table 8 and using the T-student and the Mann Whitney methods, we found no statistically significant relationship between the patient gender and the quality of life with a P-value of 0.6. Nonetheless, female patients had a little worse quality of life score with an average of 2.43 (SD 1.02) compared to male individuals who scored an average of 2.52 (SD 1.17).

**Table 8:** The average quality of life according to gender.

Gender	Number	Average	SD	P-value
Male	57	2.52	1.17	0.6
Female	45	2.43	1.02	

On the other hand, we discovered that the patient age played a statistically significant role in the quality of life with a P-value of 0.01 using the ANOVA and the Kruskal – Wallis methods which indicated that the age group of 20–39 years of age has suffered the worst quality of life with an average of 2.1 (SD 0.7). The quality of life then improved as the patient aged to reach an average of 4.4 (SD 0.6) in individuals older than 60 years of age. As shown in Table 9.

**Table 9:** The average quality of life according to age groups.

Age groups	Number	Average	SD	P-value
Less than 20	2	2.4	0.9	0.01
20–29	40	2.1	0.7	
30–39	31	2.5	1.2	
40–49	19	2.4	1.1	
50–59	7	3	1.4	
More than 60	3	4.4	0.6	

## 4 Discussion

We studied patients with anogenital warts presenting to the biggest hospital in the city of Latakia which is the habitat of around 1.4 million Syrian inhabitants.

The EQ-5D scores in our study showed evidence of extreme levels of anxiety and depression in more than half of the sample and a moderate effect in around a quarter of the sample. A noticeable impact was seen in 31% of participants describing moderate pain and discomfort and 20% indicating severe similar symptoms. This is consistent with what is expected of a patient to experience when receiving a diagnosis of an STI, particularly in a conservative community like Syria [8]. These findings are similar to what was discovered in the studies of S Woodhall et al. [8] and Géraldine Dominiak-Felden et al. [1] and the Chinese study of Ju-Fang Shi et al. [6] On the other hand, and in another study for S Woodhall et al. that recruited 895 participants, the highest loss of quality of life was seen in 16–19-year-old women and 35-44-year-old men. In addition to this, 37% of participants indicated moderate or extreme problems with anxiety and depression and 26% with pain or discomfort [7]. One study showed an impaired usual activity aspect in the findings of studying 272 Canadian patients in addition to impairments in the pain/discomfort and anxiety/depression dimensions. Drolet et al. [12] according to a study from South Korea on females, the most commonly reported problems were pain–discomfort (10.7%) and anxiety–depression (12.7%) [4].

Proving the disappearance of gender differences with younger generations, female participants in our study suffered only a slightly worse quality of life scores than males which is consistent with the fact that females feel more under pressure when being confronted with an STI diagnosis in addition to their fear of the disease future effect on their reproductive health, sexual life and being accepted by their sexual partners in a conservative society [1, 13]. The causative relationship between HPV and cervical and penile cancer has added up to the burden expected in both males and females [14, 15, 16]. The same worse effect on females was observed in the studies of Géraldine Dominiak-Felden et al [1] and Ju-Fang Shi et al. [6] and the Colombian study of Marion Pineros et al. [17].

In line with the finding in Syria, Scott A. McDonald et al. have found out that in the Netherlands, the effect on the quality of life was higher in women than in men, but the gender disparity in HPV-related disease appears to be fading. The study also predicted the share of males in the total disease burden to continue to rise soon [18].

The study of Lee T et al showed similar findings in that women with genital warts suffered a greater psychosocial impact than men [19]. The fear women had in Argentina was high in the Cancer and Treatment Domain as demonstrated in the study of Arrossi S at al [19].

Looking at the age groups, we proved that the age group of 20–29 years has suffered the worst quality of life which could be explained by the fact this age group reflects the years of highest sexual activity in our society where a visible sexually-transmitted infection drastically affects the sexual quality of life.

On the other hand, we noticed that the QoL improved as the patient aged to reach its best score in individuals older than 60 years of age reflecting their enjoyment of the best grade, followed by the age group of 50–59 years old. These findings could be explained by the fact that older patients would have better coping mechanisms to deal with their health. They might have gone through several bouts of the warts so they are more educated about it compared to patients receiving a first-time diagnosis (the younger patients). In our society, people are less interested in their sexual life as they age, in addition to the fact that the life expectancy in Syria is around 65 years of age. There reasons may stand behind the good QoL in older patients in our sample. It is worth to say the we had considerably less patients above 50 years, which is why; we can only depend of these findings in the frame of our sample.

We noticed an understandable relationship between the number of lesions and the worsening of quality of life where the lowest scores were recorded in patients with more than 7 lesions. This is the same finding seen in the studies of Ju-Fang Shi et al. with a statistically significant P value of less than 0.05 [6].

In our study, we found out that the impact on quality of life was somehow worse in patients with higher education levels which was reasonable looking at how concerned these groups of populations got when diagnosed with HPV in addition to caring for their health by reading and inquiring about this disease and its link to potential cancers. The same finding was seen in women population in the study of Marion Pineros et al. [17].

Marital status played a minimal role in the effect of disease on the QoL. To notice, single individuals suffered the worst quality of life which could easily be explained by their fear of the disease in their future relationships. For females, an added concern was put on future motherhood and childbearing.

Studying the correlation between the socioeconomic status and the QoL would have been interesting. However, we were unfortunately unable to implement it. In Syria and especially during the war, it has become extremely difficult to assess the socioeconomic status of individuals as a great percentage of the population now depends on help from humanitarian organizations or from family members abroad. Besides, asking directly about the income is socially unacceptable. For these reasons, assessing the socioeconomic status would have led to misleading information. In the study of Atallah D at al, it was found in this matter that the biggest influencers on the QoL were social and religious beliefs [19].

**Main effect:** HPV has been closely linked to cervical cancer and it could be found in around 99% of all cervical cancers [15]. In conjunction with its proven benefit to prevent genital warts, cervical and penile cancers [17, 20], the quadrivalent vaccine that includes types 6 and 11 which cause more than 90% of the infection could have a promising benefit in reducing the emotional impact of the disease in Syria [17]. This potential positive result has been already found in Australia [19].

**Future studies:** More research may be needed to better assess this project in the future. Our conversations with the patients had promising seeds that the HPV vaccine is expected to be welcomed in the Syrian community as a possible way to prevent a bothersome STI, dreadful cancer and an unfortunate effect on emotional wellbeing. The study done in the Philippines by Chanprasertpinyo used good ways in studying the steps recommended to initiate good vaccination campaign against HPV in young individuals. Education programs on HPV infection and vaccination should be included in the curriculum earlier, if possible, since primary school because the vaccine works best before the onset of sexual activity. In university students, the education programs may encourage the sexually inexperienced students to receive the vaccines, as they are still the ideal group for catch-up vaccination [19].

What is the correlation between the age effect and the indicative severity effect? Is there a significantly higher disease severity within the age group of 20–29? In addition, quality of life is also greatly influenced by social support and the socioeconomic status. We hope to see further research shedding a light on these aspects.

**Conservative culture:** Living in a conservative society largely worsened these negative feelings by blaming the patients as victims and stigmatizing them for having sexual relationships. All these factors should only drive researchers to perform bigger scale studies with a larger number of participants to better estimate our results. Other aspects that could affect the quality of life might be studied like the length of the disease course, the number of recurrences, location of lesions and the sexual activities being carried out by patients.

**Vaccination:** The HPV vaccination is strongly recommended to be part of the national vaccination campaign and this will need more international cooperation through states or investors to overcome the obstacles in importing vaccines to the sanctioned country and other related funding issues.

Lastly, we should stress the idea that the medical personnel dealing with the patients, and mainly the treating doctors, plays an essential role in comforting the patients throughout the process of examination, diagnosis and treatment. This positive bedside manner is crucial in alleviating the negative psychological suffering, especially in a society where the doctor might be the only tension-relieving point for the patients as their families and societies might regrettably reject them either temporarily or permanently.

## 5 Conclusions

Expressed either naturally or upon asking, patients with HPV-caused anogenital warts talked about feeling angry, ashamed of the disease, worthless or having lost their sexual interest partially or sometimes completely. This study proved an adverse effect on the quality of life when being diagnosed with genital warts. The populations most affected were young educated individuals, females and patients with numerous lesions.

### 5.1 Limitation

National data on this disease is missing due to the incapacity of the Ministry of Health to keep track of medical statistics nationally, especially after the war erupted in 2011. 70% of our sample aged between 20 to 39 years old which is consistent with the years of highest sexual activity in Syria.

As this is the first study in Syria to use standardized measures to estimate the quality of life in genital warts patients, we were only able to study and compare the results internally within our sample to come up with conclusions.

Due to its sensitivity and especially during the Syrian war, we were unable to establish the relationship between the socioeconomic status and the quality of life.

## Declarations

### Author contribution statement

Joud Haddad: Conceived and designed the experiments; Contributed reagents, materials, analysis tools or data; Wrote the paper.

Fouz Hasan: Conceived and designed the experiments.

Abdel Halim Roumeih: Performed the experiments.

Abdullah Omar: Analyzed and interpreted the data; Contributed reagents, materials, analysis.

### Funding statement

This research did not receive any specific grant from funding agencies in the public, commercial, or not-for-profit sectors.

### Data availability statement

Data included in article/supp. material/referenced in article.

### Declaration of interest's statement

The authors declare no conflict of interest.

### Additional information

No additional information is available for this paper.
