# Supplementary material for: The psychosocial burden of anogenital warts on Syrian patients: study of quality of life
Source: Heliyon. 2022 Jun 30;8(7):e09816. doi: 10.1016/j.heliyon.2022.e09816 (PMC9260334; doi:10.1016/j.heliyon.2022.e09816)
Supplement: Supplementary file [file mmc1.docx]

**Appendix: (the questionnaire).**

Name: Gender: Age:

Marital status: Educational level: Occupation:

Phone number: Number of lesions:

**General questionnaire**

| Dimension | Grade of problem | Answer |
| --- | --- | --- |
| Mobility | I have no problems in walking about |  |
|  | I have some problems in walking about |  |
|  | I am confined to bed |  |
| Self-care | I have no problems with self-care |  |
|  | I have some problems washing or dressing myself |  |
|  | I am unable to wash or dress myself |  |
| Usual activities | I have no problems with performing my usual activities |  |
|  | I have some problems with performing my usual activities |  |
|  | I am unable to perform my usual activities |  |
| Pain/discomfort | I have no pain or discomfort |  |
|  | I have moderate pain or discomfort |  |
|  | I have extreme pain or discomfort |  |
| Anxiety/depression | I am not anxious or depressed |  |
|  | I am moderately anxious or depressed |  |
|  | I am extremely anxious or depressed |  |

**Specific questionnaire:**

|  | Always  1 | Almost always  2 | Sometimes  3 | Rare  4 | Never  5 |
| --- | --- | --- | --- | --- | --- |
| I am afraid that the lesions won’t disappear. |  |  |  |  |  |
| I am anxious to know whether I am going to recover from the infection for good. |  |  |  |  |  |
| I worry about whether the warts will get worse or whether there will be some complications. |  |  |  |  |  |
| My state of mind is upset (anxiety, depression, sadness, uneasiness…). |  |  |  |  |  |
| I feel more insecure. |  |  |  |  |  |
| Knowing that I have the illness affects me in my daily life. |  |  |  |  |  |
| My sexual drive has decreased. |  |  |  |  |  |
| I feel worried during sexual relations. |  |  |  |  |  |
| I avoid sexual relations. |  |  |  |  |  |
| My sexual relations have decreased in quality and/or frequency. |  |  |  |  |  |
